# Multi-Modal Tightly Coupled Robust Pose Estimation for Mobile Robots in Complex Degraded Scenarios

**DOI:** 10.3390/s26144485

**Published:** 2026-07-15

**Authors:** Huating Tian, Tao Li

**Affiliations:** 1 School of Mechanical and Electrical Engineering, Kunming University of Science and Technology, Kunming 650500, China; sunny_tht@163.com; 2 KSEC Intelligent Technology Co., Ltd., Kunming 650500, China

**Keywords:** mobile robot, pose estimation, iterated error-state Kalman filter (iESKF), multi-modal tight coupling, adaptive robustness, Lie group manifold

## Abstract

Current multi-modal pose estimation methods often suffer from severe localization divergence and prohibitive computational overhead when confronted with extreme scenarios such as sudden illumination variations, geometric degeneracy, and wheel slippage. To address these critical challenges, this paper presents a tightly coupled multi-modal pose estimation algorithm for mobile robots utilizing adaptive robust manifold filtering. First, a pre-integration-driven iterated error-state Kalman filter (iESKF) is formulated on the Lie group manifold to eliminate redundant re-integration workloads. Second, the Mahalanobis distance chi-square test and M-estimation are introduced to adaptively isolate non-Gaussian heavy-tailed noise caused by perception degradation. Finally, a perception health quantification system and a smooth degradation state machine are designed to handle concurrent perceptual blindness and wheel slippage. Experimental results demonstrate that the algorithm takes an average of only 12.8 ms per frame on edge computing platforms. Under severely degraded and composite environments, the algorithm limits the typical end-to-end closed-loop drift to 1.37 m (with a statistical average of 1.24 m) over a 100-m trajectory, translating to a relative translation error (RTE) of approximately 1.2% to 1.4%. This demonstrates an exceptional balance between high real-time efficiency and robust survivability.

## 1. Introduction

High-robustness six-degree-of-freedom pose estimation lays the foundation for autonomous navigation of mobile robots in unstructured environments [1,2]. To overcome the physical limitations of single-source perception, multi-modal tightly coupled perception, which fuses vision, Light Detection and Ranging (LiDAR), Inertial Measurement Unit (IMU), and proprioceptive kinematic information, has become increasingly important in industrial mobile robots [3,4,5]. Although multi-source fusion technologies have achieved remarkable progress under ideal working conditions, they still confront the following challenges in complex degenerate scenarios:

(1) The perceptual modal characteristics of external environments degrade significantly. Visual perception is susceptible to variations in lighting conditions and featureless regions, whereas LiDAR often exhibits longitudinal slippage and global drift in degraded scenarios with uniform geometric structures such as long straight corridors due to insufficient geometric constraints [6,7,8].

(2) Kinematic model mismatch. Introducing proprioceptive kinematic constraints can mitigate drift, but existing algorithms rely on the assumption of pure wheel rolling. When wheels slip on icy, snowy, or muddy surfaces, incorrect displacement prior information is introduced, leading to failure of the global state estimation [9,10].

(3) Non-Gaussian disturbances and computational bottlenecks. Non-Gaussian heavy-tailed noise is prone to occur under complex operating conditions [11]. The Extended Kalman Filter (EKF) is constrained by a fixed observation noise covariance matrix and the Gaussian white noise assumption, making it difficult to actively identify and isolate such anomalous outlier data. When a robot experiences rapid rotation, applying a first-order Taylor expansion to the EKF introduces significant truncation errors, compromising the filter’s consistency and ultimately causing divergence in the heading angle [12,13].

To address the aforementioned issues, mainstream multi-source fusion strategies often face a trade-off between accuracy and computational efficiency. Methodologies based on factor graph optimization (FGO), such as VINS-Mono [14] and LIO-SAM [15], achieve high global accuracy but incur substantial computational costs when processing massive amounts of asynchronous heterogeneous data.

In recent years, the Iterative Error State Kalman Filter (iESKF) has achieved a balance between computational efficiency and nonlinear accuracy through multi-step iterations in the tangent space of Lie group manifolds [7,16]. The iESKF executes state evolution within the tangent space of Lie group manifolds and implements Gauss-Newton-like multi-step iterations during the measurement update phase, successfully balancing the computational efficiency of filtering with the non-linear accuracy of optimization. However, existing manifold filtering schemes still lack regulatory mechanisms to handle sudden sensor failures when processing multi-modal asynchronous data. Therefore, introducing adaptive robust estimation concepts from robust statistics to reconstruct the weight distribution of multi-source measurement residuals is critical for achieving highly reliable navigation in complex environments.

To address the aforementioned issues, this paper proposes a multi-modal, tightly coupled, robust pose estimation method for mobile robots in complex scenarios. It overcomes the reliance of traditional graph optimization on high computational power by redeveloping a multi-modal filtering engine within a local Lie algebra space, enabling the system to smoothly degrade performance under composite interference conditions. The main contributions are as follows:

(1) Development of a pre-integration-driven multi-source manifold iterative filtering method: By integrating manifold pre-integration and an analytical first-order Jacobian compensation mechanism into the iESKF closed-loop system, this approach theoretically eliminates redundant integration computation required during high- and low- frequency asynchronous data fusion.

(2) Establishment of a unified multimodal adaptive robust update framework: By incorporating the innovation-based Mahalanobis distance chi-square test and M-estimation theory, the noise covariance matrix of anomalous measurements is dynamically expanded online to enable accurate identification and isolation of non-Gaussian outliers.

(3) Achieve smooth degradation under composite adverse environmental conditions: By employing dimensionless health metrics and a hierarchical fault-tolerant state machine, the system enables seamless fault tolerance even under combined scenarios of perceptual double-blindness and frequent persistent failures.

## 2. Related Work

Over the past decade, spatial state estimation technology has evolved from single-source dead reckoning to multi-source heterogeneous fusion, from loosely coupled to tightly coupled architectures, and from idealized Gaussian assumptions to robust non-Gaussian inference. This section provides a comprehensive overview of the current state of research both domestically and internationally across three core dimensions, while explicitly defining the scope of this study.

### 2.1. Evolution of Tightly Coupled Multi-Modal Odometry Architectures

Since Forster et al. introduced the IMU pre-integration technique on manifolds [5], optimized versions such as VINS-Mono [14] and ORB-SLAM3 [17], along with filter-based OpenVINS [18], have rapidly become research hotspots. Meanwhile, laser-inertial tightly coupled (LIO) systems like LIO-SAM [15], FAST-LIO2 [7], and Point-LIO [19] have achieved significant success in complex large-scale 3D mapping [20]. To address extreme scenarios, multimodal fusion approaches—including LVI-SAM [21], R3LIVE [22], FAST-LIVO [23], and LIC-Fusion [24]—have been developed to enhance system robustness. However, most of these methods rely on factor graph optimization, which faces computational capacity limitations on edge platforms [18]. Furthermore, optimizers struggle to produce low-latency steady-state outputs when handling dynamic disturbances.

### 2.2. Kinematic Constraints and Strategies for Coping with Extreme Degeneration

To mitigate *Z*-axis divergence during prolonged operation of pure external sensing systems, Wu et al. [3] derived joint constraints for wheel-based odometers and VIO systems; subsequent studies extended the kinematic model to rugged terrains [9]. The IMU multi-level degradation strategy proposed by Super Odometry [25] enhances system robustness in extreme environments [2].

Most existing tightly coupled odometry algorithms adopt a rigid tight coupling strategy, which relies on the assumption of ideal zero sideslip and assigns fixed low-noise priors to the optimizer. However, when the robot lifts off the ground or slips on the road surface, such rigid coupling will cause the positioning system to diverge.

### 2.3. Manifold Filtering and Non-Gaussian Robust Control

To address the computational challenges of graph optimization and the consistency issues of the EKF, Barrau and Bonnabel demonstrated the logarithmic linear properties of invariant extended Kalman filtering in error dynamics [12]; Hartley et al. successfully applied it to pose estimation for bipedal robots with sliding motion, achieving global consistency under severe disturbances [26]. Building upon Sola’s manifold derivation [13], Xu and Bai introduced the iESKF in their FAST-LIO series work [7,16], employing an approximate Gaussian Newton-based single-frame multi-step manifold iteration to mitigate linearization-induced Taylor truncation errors.

In addressing abnormal observations and non-Gaussian heavy-tailed environmental noise, the multi-source framework widely employs Huber kernel functions. Some studies have also enhanced the robustness of Kalman filtering under non-Gaussian impulse noise using robustness criteria such as maximum correlation entropy [27]. However, existing approaches still lack adaptive manifold updates in multimodal tight coupling scenarios—a key challenge addressed by exploring various kernel function properties [28].

However, it remains challenging to uniformly map high-frequency IMU pre-integration effects, vehicle degradation, and external sensing disturbances into the dynamic adaptive covariance space within a unified error-state manifold filtering framework. This paper integrates robust statistics with the classical adaptive anti-noise filtering theory in navigation measurement [29], proposing an adaptive anti-noise filtering-based method for tightly coupled multi-modal robust pose estimation in complex scenarios for mobile robots.

## 3. System Overview and Kinematics Modeling

### 3.1. Frames of Reference and Notational Conventions

To accurately describe the system state, the following coordinate systems are defined: World Coordinate System (W), IMU Body Coordinate System (B), LiDAR Coordinate System (L), and Wheel-Meter Chassis Coordinate System (O). Using the IMU coordinate system as the reference, the system’s actual state vector $x_{k}$ at time $t_{k}$ is defined as follows (Equation (1)):

(1) $$x_{k}=[(p_{k}^{W}{)}^{T},(v_{k}^{W}{)}^{T},(q_{k}^{W}{)}^{T},(b_{a,k}{)}^{T},(b_{g,k}{)}^{T}{]}^{T}$$

Including the position $p_{k}^{W}$, velocity $v_{k}^{W}$, attitude quaternion $q_{k}^{W}$ in the world coordinate system, as well as the zero offsets $b_{a,k}$ and $b_{g,k}$ of the accelerometer and gyroscope.

### 3.2. IMU Kinematics and Pre-Integration Model

The high-frequency raw measurements from the IMU—namely the angular velocity ω (Equation (2)) and acceleration a (Equation (3))—are modeled as a combination of the true physical quantities, sensor biases, and additive Gaussian white noise n:

(2) $$\omega =\omega_{true}+b_{g}+n_{g}$$

(3) $$a=R_{WB}^{T}(a^{W}-g^{W})+b_{a}+n_{a}$$

where $a^{W}$ denotes the true acceleration in the World frame, $g^{W}$ represents the gravity vector. To circumvent the computational burden of re-integrating the high-frequency IMU data upon every state update, this study adopts the IMU preintegration method on manifolds. Within a given time interval [i,j], the continuous-time kinematic differential equation of the 15-dimensional error-state minimally parameterized vector $\delta x=[\delta p^{T},\delta v^{T},\delta \theta^{T},\delta b_{a}^{T},\delta b_{g}^{T}{]}^{T}\in R^{15}$ can be formulated as (Equation (4)):

(4) $$\dot{\delta x}=F_{c}\delta x+G_{c}n$$

where $n={\left[\begin{array}{cccc} n_{a}^{T} & n_{g}^{T} & n_{ba}^{T} & n_{bg}^{T} \end{array}\right]}^{T}\in R^{12}$ represents the composite continuous-time noise vector; $n_{a}\in R^{3}$ is the white noise of the accelerometer measurement, $n_{g}\in R^{3}$ is the white noise of the gyroscope measurement, $n_{ba}\in R^{3}$ is the driving noise of the accelerometer bias random walk, $n_{bg}\in R^{3}$ is the driving noise of the gyroscope bias random walk.

Assuming that the error on the attitude manifold is defined adhering to the right-multiplication perturbation model, let $R_{WB}$ denote the rotation matrix transforming from the IMU body frame (B) to the World reference frame (W). Based on the first-order Taylor expansion truncation of the non-linear kinematic equations at the current nominal trajectory point, the analytical structures of the continuous-time state transition Jacobian matrix $F_{c}\in R^{15\times 15}$ and the noise distribution mapping matrix $G_{c}\in R^{15\times 12}$ are derived as (Equations (5) and (6)):

(5) $$F_{c}=\left[\begin{array}{ccccc} 0_{3\times 3} & I_{3\times 3} & 0_{3\times 3} & 0_{3\times 3} & 0_{3\times 3}\\ 0_{3\times 3} & 0_{3\times 3} & -R_{WB}[\hat{a}{]}_{\times } & -R_{WB} & 0_{3\times 3}\\ 0_{3\times 3} & 0_{3\times 3} & -[\hat{\omega }{]}_{\times } & 0_{3\times 3} & -I_{3\times 3}\\ 0_{3\times 3} & 0_{3\times 3} & 0_{3\times 3} & 0_{3\times 3} & 0_{3\times 3}\\ 0_{3\times 3} & 0_{3\times 3} & 0_{3\times 3} & 0_{3\times 3} & 0_{3\times 3} \end{array}\right]$$

(6) $$G_{c}=\left[\begin{array}{cccc} 0_{3\times 3} & 0_{3\times 3} & 0_{3\times 3} & 0_{3\times 3}\\ -R_{WB} & 0_{3\times 3} & 0_{3\times 3} & 0_{3\times 3}\\ 0_{3\times 3} & -I_{3\times 3} & 0_{3\times 3} & 0_{3\times 3}\\ 0_{3\times 3} & 0_{3\times 3} & I_{3\times 3} & 0_{3\times 3}\\ 0_{3\times 3} & 0_{3\times 3} & 0_{3\times 3} & I_{3\times 3} \end{array}\right]$$

In the above derivation, the operator $[\cdot {]}_{\times }$ denotes an isomorphic mapping from a three-dimensional real vector to a 3 × 3 skew-symmetric matrix; the parameters used to construct the state transition matrix are unbiased measurements (${\hat{a}=a}^{m}-b_{a}$ and ${\hat{\omega }=\omega }^{m}-b_{g}$).

### 3.3. Multimodal Observation Model

(1) Visual Reprojection Observation Model

Define the camera coordinate frame as C. The rigid extrinsic calibration parameters between the camera and the IMU body frame B are denoted as $T_{BC}=\{R_{BC},p_{BC}\}$. Utilizing the current predicted system state, the geometric continuous transformation involved in projecting the j-th static landmark point from the World frame into the local camera frame $C_{k}$ as 3D coordinates $p_{l,j}^{C_{k}}=[X_{c},Y_{c},Z_{c}{]}^{T}$ is expressed as (Equation (7)):

(7) $$p_{l,j}^{C_{k}}=R_{BC}^{T}((R_{k}^{W}{)}^{T}(p_{l,j}^{W}-p_{k}^{W})-p_{BC})$$

According to the pinhole camera model, the tightly coupled observation equation is formed between the theoretical projection of this 3D spatial point onto the normalized image plane and the pixel measurement value $z_{cam,j}$ (Equation (8)):

(8) $$z_{cam,j}=\left[\begin{array}{c} f_{u}\frac{X_{c}}{Z_{c}}+c_{u}\\ f_{v}\frac{Y_{c}}{Z_{c}}+c_{v} \end{array}\right]+v_{cam}$$

where $f_{u},f_{v}$ and $c_{u},c_{v}$ are the focal lengths and principal point coordinates within the camera intrinsic matrix, respectively; $v_{cam}$ represents the pixel-level composite measurement noise in the feature extraction process.

Under nominal operating conditions, $v_{cam}$ is assumed to follow a zero-mean Gaussian distribution. However, when industrial mobile robots operate under abrupt lighting changes or direct backlighting, visual residuals significantly deviate from this Gaussian assumption, exhibiting heavy-tailed non-Gaussian characteristics. This is also the primary motivation behind introducing the Mahalanobis distance chi-square test and the M-estimation adaptive covariance soft isolation in this paper.

(2) Wheeled Chassis Kinematic Observation Model

To accommodate different types of chassis for industrial mobile robots, a unified kinematic abstraction model is established. Non-holonomic mobile bases—such as differential-drive, single-steer, or Ackermann steering—can ultimately be transformed into linear velocities $v_{x},v_{y}$ and the angular velocity $\omega_{z}$ expressed in the chassis frame. Taking the extrinsic parameters $T_{O}^{B}$ between the wheels and the IMU into account, the observation equation of the wheel odometry is defined as (Equation (9)):

(9) $$z_{wheel}=h_{wheel}(x_{k})+v_{wheel}$$

The velocity of the IMU center in the World frame, $v_{k}^{W}$, cannot be directly projected to represent the chassis velocity due to the lever-arm effect during rotational motion. Let the extrinsic spatial transformation from the IMU body frame (B) to the chassis frame (O) be defined by the rotation matrix $R_{BO}$ (from O to B) and the translation vector $p_{BO}^{B}$ (the position of the chassis center relative to the IMU center, expressed in the B frame). Utilizing the unbiased IMU angular velocity measurement $\omega^{B}=\omega -b_{g}$, the true velocity of the chassis center projected into the local chassis frame (O) is kinematically derived as (Equation (10)):

(10) $$v^{O}=[v_{x}^{O},v_{y}^{O},v_{z}^{O}{]}^{T}=R_{BO}^{T}((R_{k}^{W}{)}^{T}v_{k}^{W}+\lfloor \omega^{B}\rfloor_{\times }p_{BO}^{B})$$

where $(R_{k}^{W}{)}^{T}$ transforms the velocity from the World frame to the IMU body frame, and $\lfloor \cdot \rfloor_{\times }$ denotes the skew-symmetric matrix operator. Based on the pure rolling assumption, the non-holonomic mobile base is constrained from moving laterally or vertically. Thus, the Non-Holonomic Constraint (NHC) virtual observation equation is rigorously formulated as (Equation (11)):

(11) $$\left[\begin{array}{c} v_{y}^{O}\\ v_{z}^{O} \end{array}\right]=\left[\begin{array}{c} 0\\ 0 \end{array}\right]+n_{nhc}$$

where $n_{nhc}∼N(0,R_{nhc})$ represents the 2×1 zero-mean Gaussian noise vector characterizing the physical relaxation of this constraint.

(3) LiDAR Pose Measurement Model

Similarly, the LiDAR scan is matched with the local map based on ICP or NDT algorithms to obtain the relative pose transformation or direct global pose observations. After transforming these measurements to the IMU coordinate frame, the LiDAR observation equation is established as (Equation (12)):

(12) $$z_{lidar}=h_{lidar}(x_{k})+v_{lidar}$$

## 4. Adaptive Robust Multi-Source Manifold Iterative Filtering Algorithm

This paper studies an adaptive robust iterative error-state Kalman filtering algorithm based on Lie group manifolds. It adopts IMU preintegration to construct the state prediction module and relieve the computational burden brought by high-frequency sensor data. By combining M-estimation with Mahalanobis distance chi-square test, an adaptive adjustment mechanism for multi-source observations is established. Functionally, the proposed AR-iESKF serves as a local sequential state estimator rather than a global optimization solution. This architectural choice prioritizes deterministic ultra-low latency and real-time survivability on edge computing platforms over global offline map consistency.

### 4.1. Pre-Integration-Driven iESKF State Propagation Framework

This section performs state parameterization based on Lie group manifolds and adopts pre-integration to realize state propagation.

#### 4.1.1. Error-State Parameterization on Lie Group Manifolds

In the manifold filtering scheme, the robot state lies on a topologically smooth composite manifold M, and the overall state vector x∈M. Among them, the attitude component belongs to the orthogonal group SO(3), the position and velocity belong to the Euclidean space $R^{3}$, and the sensor bias terms belong to vector spaces.

Using error-state parameterization, the complex nonlinear evolution is transferred to the tangent space (Lie algebra space so(3)) containing the identity element for processing. The relationship between the true state $x_{true}$ and the nominal state $\hat{x}$ is defined as (Equation (13)):

(13) $$x_{true}=\hat{x}⊞\delta x$$

where $\delta x\in R^{15}$ denotes the minimally parameterized error vector, encompassing the attitude error $\delta \theta \in R^{3}$, position error $\delta p\in R^{3}$, velocity error $\delta v\in R^{3}$, and the gyroscope and accelerometer bias errors $\delta b_{g}$, $\delta b_{a}\in R^{3}$, ⊞ represents the generalized addition (or retraction) on the Lie group manifold. Because operations like adding a 3D error vector to a 3D rotation matrix are mathematically invalid in standard Euclidean space, this operator maps the error state from the local tangent space (Lie algebra) back onto the non-linear global manifold (Lie group), as summarized in Table 1.

#### 4.1.2. Preintegration-Driven High-Frequency Prior State Propagation

In a tightly coupled system, the sampling frequency of the IMU is much higher than that of cameras or LiDARs. Re-integrating the raw IMU data for each observation will lead to excessive computational costs, and there also exists time asynchrony among different sensors. In this study, pre-integration is adopted as the high-frequency prior input of the iESKF. The core of manifold pre-integration is to process the inertial measurements between two adjacent measurement moments $t_{i}$ and $t_{k}$ based on the Lie group kinematics model, and derive the relative increments $\Delta R_{ik}$, $\Delta v_{ik}$ and $\Delta p_{ik}$ that are independent of the initial pose. Its discrete-time accumulation process is expressed as follows:

Attitude Preintegration (Equation (14)):

(14) $$\Delta R_{ik}=\Delta R_{i,k-1}Exp((\omega_{k}-b_{g,i})\Delta t)$$

Velocity Preintegration (Equation (15)):

(15) $$\Delta v_{ik}=\Delta v_{i,k-1}+\Delta R_{i,k-1}(a_{k}-b_{a,i})\Delta t$$

Position Preintegration (Equation (16)):

(16) $$\Delta p_{ik}=\Delta p_{i,k-1}+\Delta v_{i,k-1}\Delta t+\frac{1}{2}\Delta R_{i,k-1}(a_{k}-b_{a,i})\Delta t^{2}$$

where $\omega_{k}$ and $a_{k}$ represent the raw measurement data of angular velocity and acceleration from the IMU sensor at timestamp $t_{k}$ respectively; $b_{g,i}$ and $b_{a,i}$ denote the posterior estimated values of the biases locked by the system at the initial preintegration timestamp $t_{i}$.

During the measurement update and multi-step iteration phases of the adaptive iESKF, the posterior estimates of the sensor biases continuously yield minor correction components ($\delta b_{g}$ and $\delta b_{a}$). To reduce computing power consumption, this study simultaneously derives and retains the analytical Jacobian matrix of pre-integrated quantities with respect to sensor biases during the integration evolution process. Since the variations in velocity and position reside within the linear Euclidean vector space $R^{3}$, the zero bias correction can be directly expressed using a first-order Taylor expansion (Equations (17) and (18)):

(17) $$\Delta v_{ik}(b_{g}+\delta b_{g},b_{a}+\delta b_{a})\approx \Delta v_{ik}({\bar{b}}_{g},{\bar{b}}_{a})+J_{b_{g}}^{\Delta v}\delta b_{g}+J_{b_{a}}^{\Delta v}\delta b_{a}$$

(18) $$\Delta p_{ik}(b_{g}+\delta b_{g},b_{a}+\delta b_{a})\approx \Delta p_{ik}({\bar{b}}_{g},{\bar{b}}_{a})+J_{b_{g}}^{\Delta p}\delta b_{g}+J_{b_{a}}^{\Delta p}\delta b_{a}$$

For the rotation matrix residing on the non-linear manifold, its first-order partial derivative according to the multiplicative exponential mapping is formulated as (Equation (19)):

(19) $$\Delta R_{ik}(b_{g}+\delta b_{g})\approx \Delta R_{ik}(b_{g})Exp(J_{b_{g}}^{\Delta R_{ik}}\delta b_{g})$$

According to the manifold calculus, the first-order partial derivative of the rotation increment with respect to the gyroscope bias, $J_{b_{g}}^{\Delta R_{i,k}}\in R^{3\times 3}$, satisfies the discrete-time equation in (Equation (20)):

(20) $$J_{b_{g}}^{\Delta R_{i,k}}=-\sum_{m=i}^{k-1}\left(\Delta R_{m+1,k}^{T}J_{r}^{m}\Delta t\right)$$

where $J_{r}^{m}$ represents the right Jacobian of the 3D rotation vector within the local interval of the Lie algebra. Through first-order Taylor error compensation, the posterior updates of the biases are decoupled from the re-integration binding of high-frequency raw data, thereby eliminating the computational bottlenecks of multi-step iterations.

To guarantee the long-term numerical stability of the analytical Jacobian compensation (Equations (17)–(19)), the proposed iESKF architecture utilizes a high-frequency error-state reset mechanism. By actively updating the nominal biases ($b_{g},b_{a}$) and strictly resetting the corresponding error-state increments ($\delta b_{g},\delta b_{a}$) to zero after each multi-modal measurement update (typically at 10~50 Hz), the Taylor expansion is perpetually evaluated over a microscopic time horizon (Δt≈0.02∼0.1 s). This periodic state reset ensures that the expansion evaluation point remains in the extreme local vicinity of the true operating point, mathematically bounding the truncation error and preventing super-linear error accumulation regardless of the global trajectory length.

#### 4.1.3. Manifold Propagation of the State Covariance Matrix

To meet the computational requirements of the edge computing platform, explicit approximation methods based on first-order Taylor series truncation or precise numerical integration methods based on the fourth-order Runge–Kutta (RK4) method are adopted to discretize and map the continuous-time model of (Equation (4)) within the sampling period Δt. This derives the discrete-time state transition matrix $\Phi_{k}$ (Equation (21)) and the discretized system noise covariance matrix $Q_{k}$ (Equation (22)):

(21) $$\Phi_{k}\approx I_{15\times 15}+F_{c}\Delta t$$

(22) $$Q_{k}\approx (G_{c}Q_{continuous}G_{c}^{T})\Delta t$$

where $Q_{continuous}\in R^{12\times 12}$ represents the continuous-time noise power spectral density matrix. The prior propagation equation for the error-state covariance matrix P is formulated as (Equation (23)):

(23) $$P_{k+1}=\Phi_{k}P_{k}\Phi_{k}^{T}+Q_{k}$$

For the error-state space $R^{15}$,the state transition matrix $\Phi_{k}$ characterizes the cross-correlation among the attitude, velocity, and biases. It is noteworthy that the proposed algorithm introduces a random walk model during the propagation phase to capture the time-varying characteristics of the sensor biases, which enables the covariance matrix to dynamically capture fluctuations in IMU stability.

### 4.2. Multi-Source Manifold Residual Mapping and Adaptive Robust Update Mechanism

In complex industrial scenarios, camera overexposure, LiDAR false positives, and wheel slipping are prone to injecting non-Gaussian outliers into the system. To address this issue, this section establishes a dual-layer robust update framework. This framework hard-rejects anomalous outliers via a chi-squared test based on the Mahalanobis distance, and concurrently isolates moderate noise while adaptively adjusting the covariance matrix by integrating M-estimation theory.

#### 4.2.1. Unified Lie Algebra Mapping of Heterogeneous Multi-Modal Residuals

To achieve the objective of fusing visual features, LiDAR surfels, and wheel kinematics constraints within a unified framework, it is essential to establish a mathematically and physically unified representation for residuals. Here, the holistic observation models are abstracted into a manifold mapping function (Equation (24)). The linearized residual equation is subsequently constructed at the current linearization estimation point $x_{k}^{\kappa }$ (Equation (25)):

(24) $$z_{k}=h(x_{k})+v_{k}$$

(25) $$z_{k}\approx h(x_{k}^{\kappa })+H_{\kappa }\delta x+v_{k}$$

The multi-step manifold iterative process is mathematically equivalent to solving a non-linear penalized cost function J(δx) that minimizes the dual Mahalanobis distances:

(26) $$J(\delta x)=\frac{1}{2}\|\delta x{\|}_{P_{k|k-1}^{-1}}^{2}+\frac{1}{2}\|z_{k}-h(x_{k}^{\kappa })-H_{\kappa }\delta x{\|}_{R^{-1}}^{2}$$

where the Jacobian matrix $H_{\kappa }\in R^{m\times 15}$ (m is the dimension of the current measurement residual) is derived through manifold differentiation as (Equation (27)):

(27) $$H_{\kappa }={\left.\frac{\partial h(x_{k}^{\kappa }⊞\delta x)}{\partial \delta x}\right|}_{\delta x=0}$$

This mapping process not only updates the 6-DoF pose of the system, but also implicitly corrects the high-frequency IMU bias drift and velocity errors defined in the $R^{15}$ space based on the cooperative feedback of multi-source residuals.

#### 4.2.2. Mahalanobis Chi-Square Anomaly Detection and IRLS Covariance Adaptive Soft-Isolation

To guarantee computational efficiency and enable feature-level outlier isolation, the Mahalanobis distance is evaluated marginally (point-wise) for each independent feature, bypassing the intractable $O(m^{3})$ inversion of the fully coupled innovation covariance matrix. Assuming conditional independence among measurements, the measurement noise covariance R is block-diagonal. For the i-th feature, the marginal normalized squared Mahalanobis distance $D_{M,i}^{2}$ of its local prior measurement innovation $r_{0,i}$ is calculated for the chi-squared test:

(28) $$D_{M,i}^{2}=r_{0,i}^{T}(H_{0,i}P_{k|k-1}H_{0,i}^{T}+R_{i}{)}^{-1}r_{0,i}$$

where the core denominator component is the marginal prior innovation covariance matrix. Because $H_{0,i}P_{k|k-1}H_{0,i}^{T}+R_{i}$ is at most a 3×3 block matrix, its inversion requires merely O(1) operations. Evaluating this across all m measurement dimensions restricts the anomaly detection complexity to a strictly linear $O(m\cdot M^{2})$. Under the nominal Gaussian noise assumption, $D_{M,i}^{2}$ should theoretically adhere strictly to a chi-squared distribution $\chi^{2}(m_{i})$ with degrees of freedom equal to the local measurement dimension $m_{i}$. In practice, the significance level α is empirically set to 0.05 (corresponding to a 95% confidence interval), a widely adopted standard in robust filtering that optimally balances outlier rejection and the retention of valid multi-modal constraints. Guided by this defined significance level, the system deterministically computes the dynamic chi-squared threshold $T_{\chi^{2}}$ online utilizing the inverse cumulative distribution function (CDF) of the chi-squared distribution, formulated as $T_{\chi^{2}}=F^{-1}(1-\alpha ;m)$. This formulation ensures that the threshold automatically adapts to the varying measurement dimensions m of the heterogeneous multi-modal data (e.g., yielding $T_{\chi^{2}}\approx 5.991$ for m=2 visual pixel observations, and $T_{\chi^{2}}\approx 7.815$ for m=3 3D wheel kinematics). Once $D_{M}^{2}>T_{\chi^{2}}$ is detected, it indicates that a severe structural failure has occurred in the current observation. Consequently, the state machine will immediately trigger the hard-rejection logic, isolating this specific frame of data to protect the prior prediction.

However, for proprioceptive kinematic constraints, completely discarding the constraint might lead to severe system unobservability. Therefore, as explicitly governed by the state machine in Table 2, if a kinematic measurement severely breaches the chi-square threshold (e.g., during severe wheel skidding), the system bypasses hard rejection and directly triggers the Degraded Mode. It forcefully switches to the Cauchy kernel to execute aggressive soft-isolation and adaptive covariance inflation, ensuring a weak but safe continuous constraint.

**Table 2:** Quantitative switching rules and weight formulations for adaptive robust kernels.

Kernel Function	Weight Formula $w(D_{M})$	Switching Trigger Condition
Huber Kernel	$w(D_{M})=\left\{\begin{array}{c} 1,D_{M}\leq c\\ \frac{c}{D_{M}},D_{M}>c \end{array}\right.$	Default Mode: Applied when the multi-source health metrics (defined in Table 3) of the corresponding sensor remain within nominal bounds.
Cauchy Kernel	$w(D_{M})=\frac{1}{1+(D_{M}/c{)}^{2}}$	Degraded Mode: Triggered strictly when a specific modal health metric breaches safety boundaries (e.g., tracked features N<15 or chassis kinematic violation).

**Table 3:** Health metrics and degradation trigger conditions for different sensors.

Sensor	Health Metric	Degradation Trigger Condition	Adaptive Compensation
LiDAR	Minimum eigenvalue of the information matrix $\lambda_{min}$	$\lambda_{min}<\tau$ (Vanishing of constraints in critical directions)	Activate non-holonomic constraints (NHC) and consistency check for point cloud distribution entropy
Visual Odometry	Effective tracking of feature point count N	Number of tracked points N<15	Increase IMU preintegration weight; lock visual scale bias
Wheel Odometry	Chassis side-slip and vertical motion residuals $\left\|z_{nhc}\right\|$	Violation of kinematic consistency	Trigger skidding discrimination logic; exponentially inflate covariance

For anomalous measurements that successfully pass the chi-squared test yet still reside within the non-Gaussian heavy-tailed region, as well as for the severely degraded kinematic constraints mentioned above, direct elimination would lead to the loss of valuable weak constraint information. To unify the mathematical handling of these scenarios, the proposed algorithm deeply integrates the M-estimation theory from robust statistics into the iterative solution phase within the local tangent space. Under the Iteratively Reweighted Least Squares (IRLS) framework, the κ-th manifold iteration calculates a diagonal weight penalty matrix $W_{\kappa }=diag(w_{i})$ based on the current iterated measurement residual $r_{\kappa }$, utilizing robust kernel functions. The algorithm provides two types of kernel functions adapted to different working conditions, as summarized in Table 2.

The standard tuning parameter for the Huber kernel is c=1.345, and the recommended threshold for the Cauchy kernel is c=2.0. The measurement noise covariance matrix R undergoes an adaptive inflation calculation as follows Equation (29):

(29) $${\bar{R}}_{\kappa }=RW_{\kappa }^{-1}$$

To eliminate heuristic ambiguity, the transition between the Huber and Cauchy kernels is not based on arbitrary scalar evaluations, but is explicitly governed by the continuous multi-source perceptual health metrics defined in Section 4.3.1 (Table 3). Under normal operation, the system defaults to the Huber kernel to smoothly suppress mild non-Gaussian noise while preserving valid weak constraints. However, when the continuous health metric of a specific sensor explicitly violates its predefined degradation boundary, the state machine preemptively flags the corresponding modality as sub-healthy. At this deterministic trigger point, the algorithm dynamically overrides the robust weighting function for that specific sensor stream and forcefully switches it to the Cauchy kernel. Unlike the Huber kernel, which maintains a linear penalty for large errors, the Cauchy kernel features a strictly redescending derivative. This explicit mechanism proactively provides aggressive soft-isolation for severely contaminated gradients before they breach the chi-square hard-rejection boundary. This dual-layer architecture explicitly balances the statistical trade-offs between outlier rejection and information preservation: the Chi-square test prevents catastrophic divergence by hard-rejecting severe structural anomalies at the risk of temporary constraint loss, while M-estimation maintains essential, albeit weak, system observability through the soft-isolation of partially degraded measurements.

### 4.3. Smooth Degradation and State Reconstruction Under Composite Degraded Conditions

Mobile robots often encounter the phenomenon of perceptual double blindness. Under such conditions, traditional tightly coupled algorithms are highly prone to catastrophic failure or system collapse under these conditions due to the rank deficiency of the Jacobian matrices [30]. To address this challenge, this section discusses how to achieve smooth degradation of the system based on the quantification of perceptual health and state machine logic.

#### 4.3.1. Multi-Source Perceptual Health Quantification and Dynamic Weight Allocation

To actively perceive environmental alterations, a set of health quantification metric frameworks based on information geometry is established herein. Because the measurement units of vision (pixels), LiDAR (meters), and wheel odometry (meters per second) exhibit significant discrepancies, directly mixing multi-modal observation equations within the error-state space would result in a numerical scale imbalance of the Jacobian gradients. Consequently, when decomposing the information matrix, the extracted eigenvalues lose their physical significance for reflecting geometric degradation. Table 3 lists the health metrics and degradation trigger conditions for different sensors.

To address this dimensional conflict, prior to performing singularity analysis and eigenvalue decomposition on the all-source observation information matrix $\Omega_{obs}=H^{T}R^{-1}H$, a prior diagonal normalization scale matrix S defined in the $R^{15}$ state space is introduced. Its diagonal elements are strictly composed of the prior nominal variances of each error-state dimension. Here, the diagonal of the prior covariance matrix driven by the current-moment preintegration is directly extracted, formulated as (Equation (30)):

(30) $$S=diag(P_{k|k-1})$$

Through a congruence transformation, the matrix is non-dimensionalized to obtain the scale-normalized evaluation information matrix ${\tilde{\Omega }}_{obs}$ (Equation (31)).

(31) $${\tilde{\Omega }}_{obs}=S^{1/2}\Omega_{obs}S^{1/2}$$

The matrix $S^{1/2}$ acts as a preconditioner to eliminate heterogeneous dimensions, enabling the information matrix to be mapped into a dimensionless geometric space. Subsequently, eigenvalue decomposition is performed on ${\tilde{\Omega }}_{obs}$ within the homogeneous scale space. If the minimum eigenvalue $\lambda_{min}$ is monitored to be lower than the pre-set geometric degradation boundary threshold τ, the system considers that the filter has suffered a rank deficiency along the physical space direction of the corresponding eigenvector, the system state machine will preemptively trigger a smooth degradation strategy.

This transforms the system from traditional data-driven operation to active state perception. It isolates risks in advance when sensors are in a sub-health state and prevents erroneous gradient information from interfering with the manifold update process.

#### 4.3.2. Fault-Tolerant Strategy of State Machine Under Sensing Failure and Slipping Conditions

By establishing fault-tolerant state machines at different levels, we ensure the reliability and continuous operational performance of the positioning system under complex interference environments. The state machine switches among three modes: full-source tight coupling, kinematics enhancement and pure inertial protection.

Constraint Enhancement and Weight Transfer Layer: If external perception manifests a weak constraint condition, the state machine immediately suspends the estimation of IMU biases.

Slippage Discrimination and NHC Adaptive Isolation Layer:

(1) When the performance of vision or laser systems degrades without slippage occurring, NHC is adopted to suppress the offset of the *Z*-axis and lateral directions.
(2) If wheel slip is detected, increase the measurement noise covariance of the wheel speed sensor and NHC. At this time, only IMU preintegration and other normal external sensors need to be used.

Stationary Detection and State Reconstruction: When displacement constraints are lost, the system combines chassis control commands with IMU characteristics to perform stationary detection and further activate the zero-velocity correction algorithm [31]. Introducing zero-velocity and zero-displacement constraints in the composite tangent space (so(3)×R12) composed of a local Lie algebra and Euclidean space can limit the drift caused by IMU noise to the sub-centimeter level.

Furthermore, to prevent rapid mode switching (chattering) caused by high-frequency signal fluctuations around the threshold in practical noisy environments, a dwell-time hysteresis mechanism is implemented within the state machine. A transition upgrading to a higher operational level (e.g., from Level 2 to Level 1) is only validated and executed if the respective health metric consistently satisfies the safety boundary for a continuous sliding window of 5 frames (approximately 0.1 s at 50 Hz). This logic ensures that momentary sensor noise spikes do not trigger unnecessary structural chattering, thereby maintaining global control stability.

### 4.4. Algorithm Process Architecture and Computational Efficiency Verification

This section outlines the comprehensive workflow of the adaptive robust filtering algorithm and demonstrates its efficiency advantages from the perspectives of both time complexity and computational resource allocation.

#### 4.4.1. Comprehensive Workflow of the Adaptive Robust Filtering Algorithm

The workflow of the algorithm follows the principles of high-frequency prediction, asynchronous caching, iterative refreshing and incremental maintenance to ensure real-time system response and stable numerical values.

High-Frequency Manifold Propagation (Thread A): The IMU drives the preintegration engine at a frequency exceeding 400 Hz, updating the nominal pose in real time within the local tangent space.

Measurement Update and Mapping Thread (Thread B): This thread is responsible for preprocessing multimodal data serialization, error-resistant measurement updates, and local map management, executed in the following serial time order:

Measurement Serialization and Initial Linearization: First, time-delay correction and extrinsic recalibration for multi-source sensors are performed to implement data buffering. A Taylor expansion is conducted on the valid observation models at the current nominal propagated pose to construct the initial observation Jacobian matrix and initial measurement innovation.

Hard Rejection (Chi-Squared Test): Based on the initial Jacobian and residuals derived in the previous step, the Mahalanobis distance of the measurement sequence is computed. By comparing it against the dynamic chi-squared threshold, outlier frames suffering from structural failure are transiently isolated and discarded, preventing anomalous data from contaminating the system prior.

Adaptive Iterative Update: For the verified measurements that pass the test, the solution is solved via the following manifold iteration:

(1) Weight Reconstruction (Soft Isolation): For abnormal measurements with non-Gaussian heavy-tailed characteristics, invoke the Huber or Cauchy kernel function according to the magnitude of residuals, recalculate the diagonal weight penalty matrix, and realize the adaptive expansion of the observation noise covariance.
(2) Multi-Round Iterative Solving: The weighted and adaptively inflated observation covariance is incorporated into the manifold optimization equation. Non-linear solving for the minimal error-state δx increment is executed cyclically for 2 to 4 iterations until the norm of the increment converges within a predefined threshold ϵ.

Manifold State Closure and Map Management: To improve the efficiency of local patch matching and map updating, an incremental dynamic octree (i-Octree) is introduced to manage local point clouds. Compared with the static kd-Tree which requires frequent global reconstruction, the i-Octree eliminates the tree reconstruction process, enhances spatial query efficiency, and reduces memory and computing power consumption [32].

#### 4.4.2. Time Complexity and Efficiency Analysis on Edge Computing Platforms

In real-time SLAM systems, computational bottlenecks often occur in the nearest neighbor search of large-scale point clouds, the evaluation of high-dimensional Jacobians, and the inversion of observation covariance matrices. A naive implementation of the multi-source measurement update would require inverting the m×m innovation covariance matrix. For hundreds of features, this would incur an intractable $O(m^{3})$ computational cost.

To guarantee real-time efficiency, our algorithm evaluates the Mahalanobis distance and M-estimation weights point-wise for each feature, bounding the robust verification complexity to a linear $O(m\cdot M^{2})$. Furthermore, during the multi-step manifold update, the Kalman gain computation utilizes the Woodbury matrix identity, algebraically projecting the dense inversion into the minimal state space to invert a fixed M×M matrix $(P_{k|k-1}^{-1}+H^{T}R^{-1}H{)}^{-1}$. This strictly confines the iterative solving complexity to $O(K(m\cdot M^{2}+M^{3}))$, ensuring robust linear scalability with respect to the measurement dimension m. Table 4 illustrates the corrected computational complexity comparison between the proposed adaptive iESKF algorithm and mainstream SLAM backend frameworks:

In Table 4, M represents the dimension of the state vector (15-DoF), m signifies the total dimension of the effective multi-modal measurements at the current step, $N_{w}$ denotes the size of the optimization sliding window, K is the number of multi-step iterations on the manifold, and $N_{p}$ refers to the scale of the local point cloud.

Through physical testing conducted on the NVIDIA Jetson Orin NX hardware platform, the average processing time per measurement update of the proposed algorithm stabilizes at approximately 12.8 ms, while the peak memory consumption remains at approximately 450 MB. To provide a transparent and honest breakdown of the computational overhead, the average execution time of each core algorithmic component per frame is detailed in Table 5.

As demonstrated in Table 5, the robust mechanisms (Mahalanobis testing and M-estimation weighting) consume approximately 3.4 ms combined (26.6% of the pipeline). Thanks to the linear scaling $O(m\cdot M^{2})$ achieved by marginal point-wise evaluation and Woodbury projection, the multi-tier robust outlier rejection process effectively isolates non-Gaussian heavy-tailed noise without dominating the runtime, satisfying the high-frequency control requirements of mobile robots.

## 5. Experimental Results

### 5.1. Experimental Platform and System Parameter Configuration

**Figure 1:**
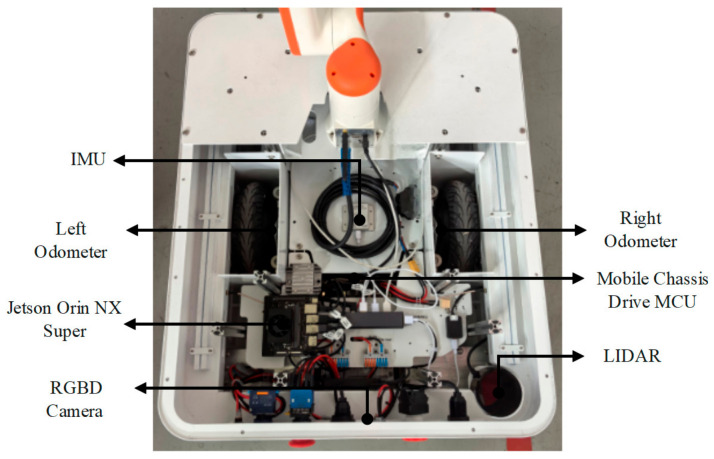
WHEELTEC S300 Pro mobile robot platform.

The experimental evaluations are conducted utilizing a WHEELTEC S300 Pro mobile robot chassis (WHEELTEC, Dongguan, China), equipped with an NVIDIA Jetson Orin NX (16 GB, NVIDIA Corporation, Santa Clara, CA, USA.) edge computing platform, as shown in Figure 1. The sensor suite deployed on the platform comprises a 16-beam LiDAR (Shenzhen RoboSense Technology Co., Ltd., Shenzhen, China), an RGB-D camera (Berxel Photonics, Shenzhen, China), and a wheel odometry system. All sensors are precisely time-synchronized via the Precision Time Protocol (PTP). Spatially, the rigid extrinsic parameters among the heterogeneous sensors are rigorously pre-calibrated offline utilizing standard spatial calibration toolboxes, providing accurate spatial priors for the tightly coupled system initialization.

To ensure algorithmic reproducibility, the core thresholds of the adaptive robust mechanism are specified as follows. The Mahalanobis chi-square test adopts α=0.05 (95% confidence interval) to balance outlier rejection and constraint preservation. The Huber kernel is set to c=1.345 for 95% asymptotic efficiency under nominal Gaussian noise, while the Cauchy kernel is empirically set to c=2.0 for aggressive soft-isolation of severe non-Gaussian disturbances. The geometric degradation threshold of the dimensionless information matrix is empirically set to $\tau =10^{-3}$, defining the critical observability margin of the sensor suite in confined spaces.

### 5.2. Accuracy Comparison Under Structured Benchmark Scenarios

**Figure 2:**
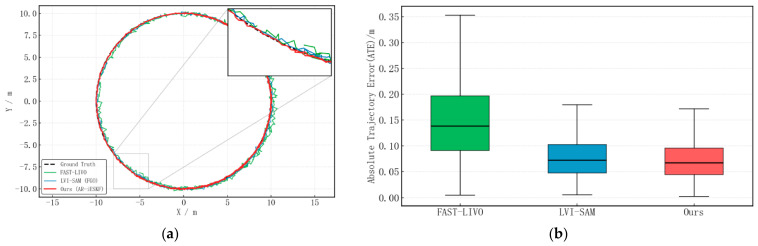
Trajectory accuracy comparison of different algorithms under structured benchmark scenarios: (**a**) Global 2D trajectory comparison curve; (**b**) Statistical absolute trajectory error (ATE) boxplot distribution.

Experimental results demonstrate that the proposed algorithm achieves a translation Root Mean Square Error (RMSE) of 0.12 m, which is highly comparable to the accuracy of LVI-SAM (0.11 m), as shown in Figure 2 and Table 6.

In this structured benchmark scenario, the ground truth reference trajectory was rigorously obtained utilizing a high-precision OptiTrack spatial motion capture system deployed in the testing facility. Crucially, the average processing time per frame of the proposed algorithm is merely 31% of that required by LVI-SAM, validating that the filtering-based scheme possesses significant computational efficiency advantages while maintaining advanced estimation accuracy.

This performance highlights a fundamental architectural trade-off. LVI-SAM achieves the highest global accuracy (0.11 m) by utilizing Factor Graph Optimization (FGO) over a sliding window, which repeatedly optimizes historical states at a significant computational cost. In contrast, our approach relies on an iESKF filtering architecture that only evaluates the current state manifold, granting it a tremendous speed advantage. The slight accuracy loss typically associated with filtering is successfully compensated for by the tight coupling of continuous wheel odometry constraints and adaptive robust kernels.

### 5.3. Robustness Verification Under Composite Degraded Conditions

#### 5.3.1. Closed-Loop Scenario Design and Objective Evaluation Benchmarks

To verify the smooth fault-tolerant performance of the proposed algorithm when encountering overlapping multi-modal physical failures, an end-to-end 100-m closed-loop test path was established using the mobile platform. Within the operational timeline t∈ [40 s, 60 s], the robot enters an artificially constructed, textureless, dim-light long corridor, where low-friction water-stained stickers are applied to the ground surface. During this phase, the mobile robot navigates at an average linear velocity of approximately 0.8 m/s. Consequently, this continuous composite degraded and slippery section spans a physical length of roughly 16 m.

For this specific test, since continuous high-precision positioning is unavailable in the degraded corridor, the ground truth was defined physically. The robot was programmed to return to a physically marked absolute origin, and the terminal Euclidean translation error was measured directly. A total of 10 repeated physical trials were conducted to ensure statistical reliability.

#### 5.3.2. Divergence Mechanism Analysis and White-Box Validation of the State Machine

**Figure 3:**
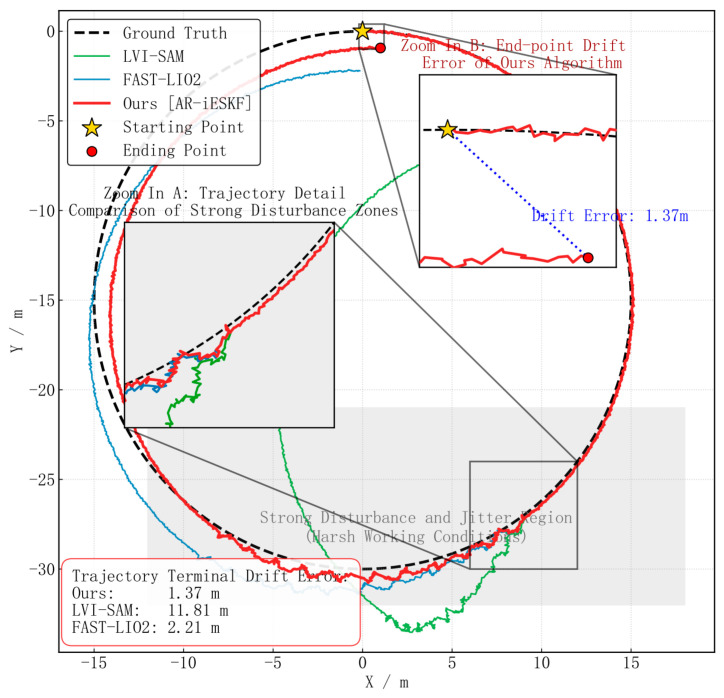
2D trajectory robust correction under degraded scenarios.

**Figure 4:**
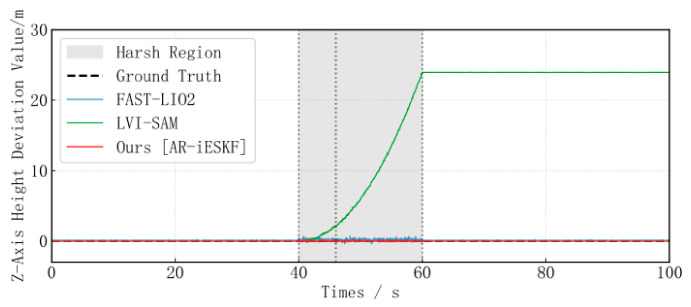
Time-space domain comparison of height-dimension distortion suppression along the *Z*-axis.

**Figure 5:**
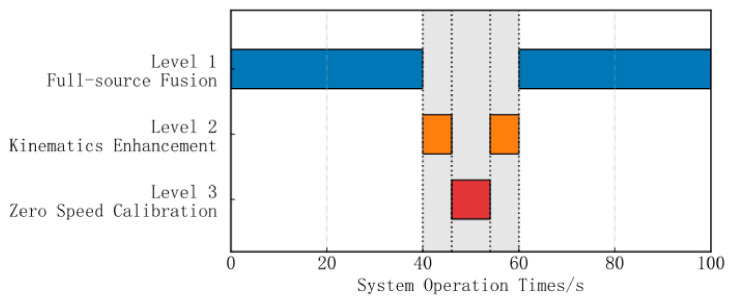
System motion state switching sequence corresponding to the hierarchical strategy of the algorithm.

The physically measured 2D closed-loop trajectory mapping, the time series of the *Z*-axis drift, and the evolution of the underlying degradation state machine are illustrated in Figure 3, Figure 4 and Figure 5, respectively.

Figure 3 presents the two-dimensional closed-loop trajectory of the proposed algorithm under a typical single extreme operating condition test, while Figure 4 illustrates the time series of the *Z*-axis drift. The end-to-end closed-loop drift is 1.37 m, and the statistically averaged drift obtained from 10 consecutive repeated trials is 1.24 m with a standard deviation of ±0.12 m. Compared with the factor graph optimization (FGO) scheme in a single closed loop (LVI-SAM, with a single-trial drift of 11.81 m and a statistical average drift of 11.70 m), the error of this algorithm is reduced by approximately 89.4% specifically under this tested composite degraded scenario. While LVI-SAM is a highly successful state-of-the-art general-purpose estimator, it inherently lacks continuous wheel kinematic constraints and explicit slippage-rejection mechanisms.

Therefore, this comparison does not imply a general superiority over LVI-SAM in nominal environments; rather, it explicitly highlights the critical necessity of integrating proprioceptive constraints and an active robust state machine to ensure survivability under extreme scenarios. It also demonstrates a better capability to handle constraint degradation when compared with the traditional LiDAR-inertial filtering scheme (FAST-LIO2, with a drift of 2.21 m). The mainstream fusion frameworks suffer from failure. At t=40 s when local spatial geometry degenerates (partial close-up in Figure 3), the trajectories exhibit severe X-Y scale divergence and outward tail flicking. In contrast, LVI-SAM, a graph optimization scheme relying on multi-modal constraints, fails to achieve X-Y closed-loop and suffers from exponential divergence along the *Z*-axis due to incorrect pre-integration priors of the optimizer under full occlusion and slippage conditions, as shown by the green curve in Figure 4.

This algorithm (red solid line) successfully passes through the 20-s blind area and docks near the physical origin. The Gantt chart of the state machine in Figure 5 provides an explanation, showing that the system’s decision-making is linked with the timeline:

(1) At t = 40 s, upon entering the corridor blind zone, the information matrix eigenvalue quantification system detects that the system observability is below the safety threshold. The state machine suspends the all-source tightly coupled state and activates the chassis non-holonomic constraints (NHC), preventing tail-wagging by introducing a zero lateral velocity expectation.
(2) At t = 46 s, during the wheel slippage phase, the Mahalanobis distance of the measurement residuals breaches the chi-squared defense line, and the state machine discards all contaminated observations. At this point, the system shunts the processing based on underlying dynamic constraints: since the IMU does not integrate a noticeable displacement at the instant of skidding, the state machine automatically downgrades to the Level 3 zero-velocity correction mode shown in Figure 5, avoiding divergence in the *Z*-axis direction.
(3) When the robot exits the blind zone and environmental features recover at t > 60 s, the state machine upgrades its dimension and resumes the all-source solution.

After multiple end-to-end experimental statistics, the average closed-loop drift of this algorithm is 1.24 m, as detailed in Table 7.

Furthermore, during this highly dynamic transition phase (40–60 s), empirical analysis indicates that the state machine is highly robust and not overly sensitive to minor noise. The transitions are deterministically driven by strictly derived statistical boundaries. Moreover, the embedded 5-frame dwell-time hysteresis mechanism (detailed in Section 4.3.2) successfully prevents high-frequency structural chattering even when sensor health metrics fluctuate near the thresholds, ensuring global control stability.

#### 5.3.3. Ablation Study: Validating the Contribution of the Adaptive Robust Mechanism

To rigorously address the concern of whether the performance improvement in composite degraded scenarios stems inherently from the proposed adaptive robust filtering algorithm rather than merely the physical addition of proprioceptive sensors, a comprehensive ablation study was conducted. We implemented a baseline denoted as “Naïve Wheel-LVI”. This ablated variant employs the exact same tightly coupled iESKF framework, fuses identical multi-modal sensory inputs (IMU, LiDAR, and Wheel Odometry), and enforces the exact same kinematic constraints (NHC) as our proposed method. However, it explicitly disables the robust modules detailed in Section 4.2.2—namely, the Mahalanobis distance chi-square test for hard-rejection and the M-estimation for covariance-adaptive soft-isolation.

The evaluation was performed under the same 40–60 s composite degraded scenario. As illustrated by the quantitative results in Table 7, the mere addition of wheel kinematics without algorithmic robustification failed to rescue the system. The Naïve Wheel-LVI baseline experienced severe trajectory divergence, yielding a closed-loop translation error of 2.84 m and a maximum *Z*-axis drift of 1.45 m.

The underlying divergence mechanism is explicitly clear: during the slippage phase on low-friction surfaces, the spinning wheels severely violate the non-holonomic pure-rolling assumption (Equations (9) and (11)) and generate false velocity constraints. Because the Naïve Wheel-LVI rigidly relies on fixed measurement covariance matrices, it blindly trusts these contaminated kinematic observations. This injected non-Gaussian, high-magnitude pseudo-gradients into the manifold optimization, permanently pulling the state estimation away from the true trajectory.

In sharp contrast, the proposed full AR-iESKF successfully contained the closed-loop error to 1.24 m. When slippage occurs, the dual-layer robust mechanism automatically recomputes the penalty weight matrix and adaptively inflates the measurement noise covariance, effectively cutting off the erroneous gradient propagation from the slipping chassis. This ablation study effectively isolates the variables and proves that the adaptive robust mechanism is the core driver ensuring the system’s smooth degradation and survivability, rather than mere hardware redundancy.

### 5.4. Robustness Analysis Against Non-Gaussian Perturbations and Wheel Skidding

**Figure 6:**
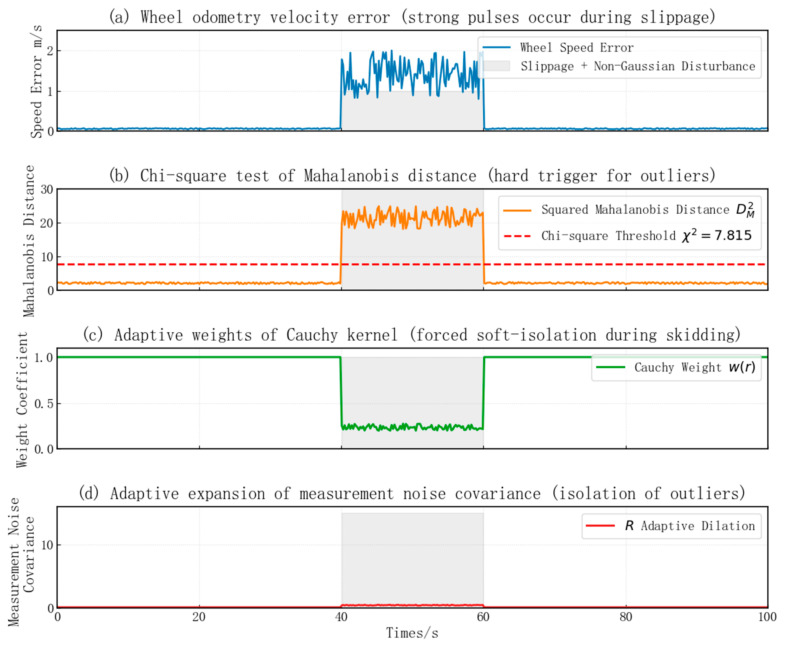
Robustness analysis against non-Gaussian perturbations and wheel skidding. (**a**) Wheel odometry velocity error with strong pulses during slippage; (**b**) Chi-square test of Mahalanobis distance; (**c**) Adaptive weights of Cauchy kernel for forced soft-isolation during skidding; (**d**) Adaptive expansion of measurement noise covariance for outlier isolation. The grey shaded area in the 40 to 60 s interval represents the wheel slippage region accompanied by non-Gaussian disturbances.

To verify the stability and fault-tolerant performance of the proposed adaptive robust mechanism under conditions of non-Gaussian interference and wheel skidding, a comparative study was conducted as shown in Figure 6.

Figure 6 illustrates the collaborative operation of the robust mechanism during the skidding interval from 40 to 60 s. As the wheel odometry error rises substantially, the Mahalanobis distance exceeds the chi-squared threshold (Figure 6b). Instead of completely discarding the kinematic constraint via hard-rejection—which could risk unobservability—the state machine actively detects the kinematic violation and triggers the Degraded Mode. Consequently, it forcefully switches to the Cauchy kernel. The robust adaptive weight drops below 0.3 (Figure 6c), and the observation covariance expands accordingly (Figure 6d). This successfully isolates the skidding error, validating the efficacy of the adaptive robust mechanism.

### 5.5. Generalizability Validation in Challenging Industrial Scenarios with Non-Gaussian Disturbances

To evaluate the generalizability of the proposed AR-iESKF algorithm in complex real-world environments, we conducted experiments in an industrial warehouse setting. To rigorously replicate extreme unmodeled non-Gaussian impacts, a rigid rectangular wooden board (3 cm thick, 100 mm wide) was anchored to the floor to serve as a standardized obstacle. Such conditions trigger severe high-frequency impulsive spikes in raw IMU measurements and periodic wheel-ground contact loss, which constitute significant violations of the standard Gaussian noise assumption and the pure-rolling kinematic constraint.

**Figure 7:**
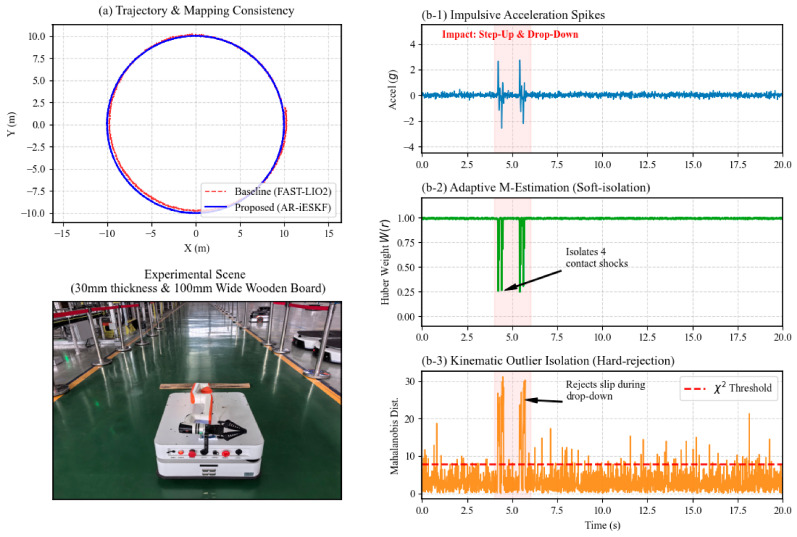
Robustness of the AR-iESKF framework in an industrial scenario utilizing a rigid wooden board. (**a**) Trajectory and mapping comparison, highlighting artifacts in the baseline caused by impulsive noise. (**b-1**–**b-3**) Decomposition of the robust filtering process: (**b-1**) impulsive acceleration spikes; (**b-2**) adaptive Huber weight adjustment (soft-isolation); and (**b-3**) Mahalanobis distance test (hard-rejection) for kinematic outliers.The pink shaded regions indicate the specific time interval during which the robot traverses the wooden board obstacle and experiences severe impulsive disturbances.

As depicted in Figure 7b-1–b-3, traversing this obstacle introduces severe impulsive disturbances. Notably, the presence of four distinct impulsive spikes in the raw IMU data (Figure 7b-1) precisely reflects the double-edge kinetic effect caused by the rigid, rectangular cross-section of the board. For a dual-axle AGV, traversing this single obstacle induces two distinct kinematic events per axle: a positive leading-edge strike (step-up) followed instantly by a negative trailing-edge drop-off (step-down). Consequently, the front axle generates a tightly coupled pair of spikes, and the rear axle generates a second pair after traversing the wheelbase distance. Traditional filtering frameworks, such as the baseline FAST-LIO2, assume Gaussian-distributed noise and lack effective outlier rejection mechanisms, inevitably incorporating these high-fidelity structural vibrations into the state estimate, leading to significant trajectory jitter and *Z*-axis drift. In contrast, our AR-iESKF employs a dual-layer robust mechanism to handle these anomalies. First, the M-estimation module utilizes a Huber loss function to dynamically reweight IMU observations when residuals exceed the predefined threshold. By adaptively scaling down the confidence of corrupted measurements (Figure 7b-2), the filter effectively isolates the impact of these impulsive IMU spikes. Simultaneously, the Mahalanobis distance chi-square test monitors the kinematic constraints. When the robot experiences momentary traction loss due to impact, the resulting non-conforming residuals are identified as outliers and strictly rejected, preventing the injection of invalid pseudo-gradients into the manifold update (Figure 7b-3).

The trajectory and mapping results are presented in Figure 7a. Compared with the baseline, which exhibits clear trajectory instability and geometric artifacts in the reconstructed map, our AR-iESKF maintains high trajectory smoothness and map consistency. Quantitative analysis, summarized in Table 8, further substantiates this performance; our method reduces the maximum *Z*-axis jitter to 0.12 m, achieving the lowest end-to-end drift among all tested baselines. This experiment explicitly demonstrates that the proposed algorithm is not only capable of operating in controlled environments but also possesses the requisite robustness for deployment in unstructured, high-impact industrial settings where non-Gaussian disturbances are prevalent.

### 5.6. Edge Computational Overhead and Real-Time Performance Evaluation

#### 5.6.1. Experimental Design and Evaluation Benchmarks

An NVIDIA Jetson Orin NX (16 GB) was selected as the hardware platform for testing. This platform operates in its maximum performance mode (with all 8 CPU cores enabled), running on the Ubuntu 22.04 LTS operating system alongside ROS2 Humble.

Three multi-source fusion localization algorithms were selected as comparative baselines, all utilizing the latest official open-source code with parameters configured to the officially recommended optimal values. FAST-LIO2 v1.0: A tightly coupled LiDAR-inertial filtering algorithm based on iESKF, which serves as a typical example of pure LiDAR filtering algorithms in terms of computational efficiency. LVI-SAM v1.0: A tightly coupled LiDAR-visual-inertial algorithm based on factor graph optimization, which is a typical representative of multi-source graph optimization algorithms. FAST-LIVO v1.0: A tightly coupled LiDAR-visual-inertial fusion filtering algorithm with the same sensor configuration as the algorithm proposed in this paper.

The test scenario is the same as the composite extreme degradation operating conditions described in Section 5.3. It includes combined interference sections featuring dimly lit corridors and wheel slippage lasting 40 to 60 s to test the entire process of the algorithm’s normal operation, degradation response and state transition. The computing power consumption and real-time performance are quantified based on three indicators: single-frame processing time, CPU usage rate and peak resident memory.

#### 5.6.2. Experimental Results and Analysis

**Figure 8:**
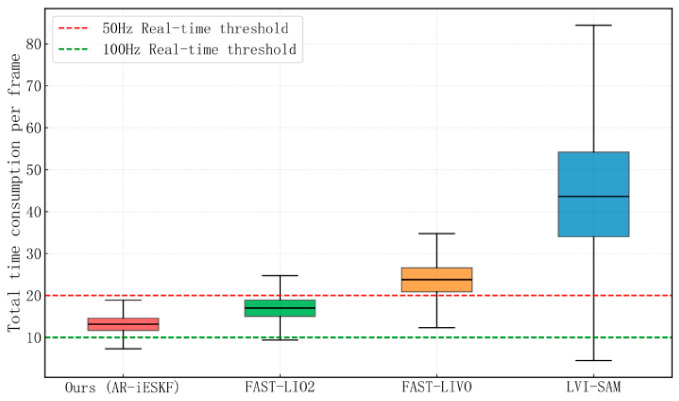
Comparison of single-frame processing time distributions among different algorithms.

The single-frame processing time directly dictates the response rate of the robot to dynamic environments. The single-frame execution time distributions for each algorithm are illustrated in Figure 8, and the quantitative statistical results are summarized in Table 9.

Thanks to the 8-core CPU’s robust parallel computing capabilities, all tested algorithms demonstrated excellent performance efficiency. The proposed algorithm requires an average of 12.8 ms per frame, with 99% of cases taking less than 18 ms, fully meeting the 50 Hz pose output requirements of industrial robots.

FAST-LIO2 belongs to the category of pure LiDAR-inertial filtering algorithms, and its efficiency remains at a relatively high level; its strength lies in the highly optimized front-end point cloud processing. In comparison, the efficiency advantage of the proposed algorithm stems from the optimization of the i-Octree graph structure regarding dynamic point cloud insertion and queries, as well as the preintegration-driven state propagation mechanism.

Within the 40-to-60-s composite degraded interval, the processing time of each algorithm increases to varying degrees: the execution time of the proposed algorithm increases by approximately 1.5 milliseconds due to the Mahalanobis distance test and the M-estimation weight recomputation; LVI-SAM exhibits the most pronounced increase in execution time because the factor graph optimization framework requires a greater number of iterations to converge when processing anomalous data.

**Figure 9:**
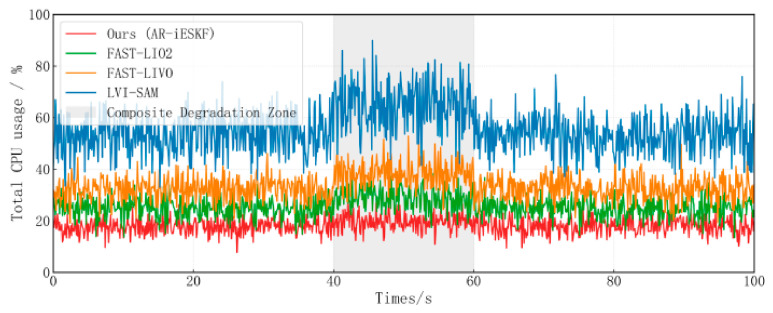
Time-series comparison of total CPU utilization rates among different algorithms.

LVI-SAM exhibits relatively lower real-time performance; however, its sliding-window-based global optimization characteristics afford superior global consistency during long-term operations, making it more suitable for high-precision mapping and offline calibration tasks in static environments. The time-series curves of the CPU utilization for each algorithm are shown in Figure 9.

The proposed algorithm achieves an average overall CPU utilization rate of 17.8%, with a peak utilization rate of 23.5%. The CPU utilization rates of FAST-LIO2 and FAST-LIVO are slightly higher than that of the proposed algorithm, with the discrepancies originating from the computational expenses of front-end point cloud feature extraction and ikd-Tree map maintenance. Because FAST-LIVO concurrently processes data from both LiDAR and visual sensors, its CPU utilization is significantly higher than that of pure LiDAR algorithms.

LVI-SAM registers an average CPU utilization rate of 53.6%, and its peak utilization can reach up to 72% within the degraded region. This occurs because the factor graph optimization must simultaneously process multiple historical states within the sliding window. However, if GPU is used to accelerate part of the matrix operations, the real-time performance will be further improved.

**Figure 10:**
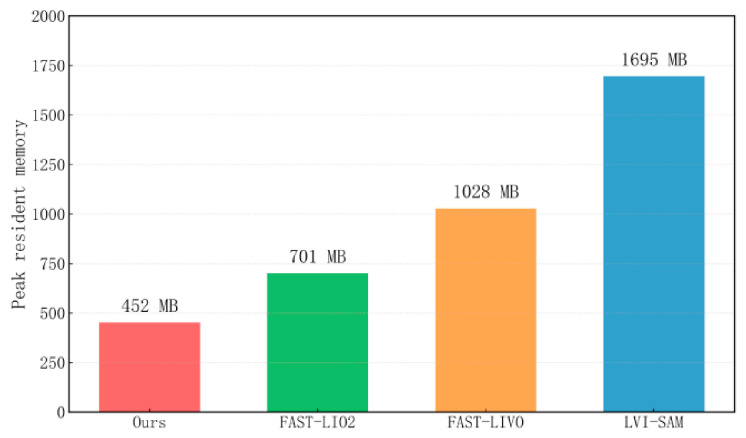
Comparison of peak resident memory consumption among different algorithms.

The peak resident memory consumption for each algorithm is illustrated in Figure 10. The peak resident memory usage of this algorithm is 452 MB, which is only 26.7% of that of LVI-SAM. Since it only maintains the current state and local map, there is no need to store historical states and marginalized data.

Because FAST-LIVO concurrently buffers LiDAR point clouds and image frame data, its memory consumption is noticeably higher than that of the pure LiDAR-based FAST-LIO2. LVI-SAM consumes the largest amount of memory, primarily because it must maintain the poses, feature points, and covariance matrices of all keyframes within the sliding window, which is the price the graph optimization framework pays to achieve global accuracy.

#### 5.6.3. Computational Efficiency Discussion

By synthesizing the theoretical time complexity analysis conducted in Section 4.4 and the physical test results in this section, the following conclusions can be drawn:

The filtering scheme possesses advantages in real-time performance: Compared to the $O(N_{w}^{3})$ time complexity of factor graph optimization, where $N_{w}$ is the sliding window size, the linear O(m) time complexity of the iESKF filtering scheme allows it to be more efficient when handling high-frequency asynchronous multi-source data. Based on preintegration-driven state propagation and the analytical Jacobian compensation mechanism, the proposed method reduces computational redundancy during multi-step iterations, constraining the single-frame state propagation time to within 1.0 ms.

The computational overhead of the robust mechanism is controllable: The Mahalanobis distance chi-squared test and the M-estimation adaptive weight mechanism designed in this study are entirely executed within the 15-dimensional error-state tangent space, yielding a computational complexity of O(m). Physical testing indicates that the extra time introduced by the robust mechanism is approximately 0.9 ms, which does not exert a noticeable impact on the global real-time characteristics of the algorithm.

Different algorithms are suited for different industrial scenarios: The proposed algorithm achieves a balance among real-time performance, memory footprint, and robustness, making it suitable for industrial mobile robots operating in complex environments with constrained computational power and high real-time requirements. FAST-LIO2 offers excellent efficiency and fair robustness, making it suitable for simple structured scenarios utilizing pure LiDAR navigation. LVI-SAM yields high global accuracy, making it suitable for high-precision mapping and offline calibration tasks in static environments.

## 6. Conclusions

To resolve the high computational overhead and localization divergence of mobile robots in extreme environments, this paper proposes a multi-modal, tightly coupled, robust pose estimation method based on adaptive robust manifold filtering. By deriving an analytical Jacobian compensation model for pre-integration in the tangent space to eliminate redundant re-integration and seamlessly combining the chi-square test with M-estimation, a dimensionless health monitoring framework and a smooth degradation strategy are established. Experimental results demonstrate that the proposed algorithm achieves excellent real-time performance and high accuracy under normal operating conditions. When confronted with superimposed working conditions such as visual degradation and wheel slippage, it maintains fault tolerance via a smooth degradation state machine. On edge computing platforms, the processing time per frame of the algorithm is only 12.8 ms. Under composite degradation conditions, the algorithm suppresses multi-dimensional distortions and realizes smooth fault tolerance. The average end-to-end closed-loop drift is controlled within 1.24 ± 0.12 m with a relative error of approximately 1.2%. Under the specifically tested composite degradation scenarios (involving concurrent perceptual double-blindness and severe wheel slippage), this represents an average error reduction of approximately 89.4% compared to the baseline LVI-SAM. Since LVI-SAM relies solely on external perception and was not originally designed to handle extreme chassis slippage, this comparative result explicitly validates the effectiveness of our proposed robust mechanism in ensuring system survivability under targeted severe conditions.

## Figures and Tables

**Table 1:** Definitions and operations of different state components.

State Component	Notation Definition	Manifold Space	Generalized Addition Operation (⊞)
Rotation matrix	R	SO(3)	$\hat{R}\cdot exp(\delta \theta^{\wedge })$
Position	p	$R^{3}$	$\hat{p}+\delta p$
Velocity	v	$R^{3}$	$\hat{v}+\delta v$
Accelerometer bias	$b_{a}$	$R^{3}$	${\hat{b}}_{a}+\delta b_{a}$
Gyroscope bias	$b_{g}$	$R^{3}$	${\hat{b}}_{g}+\delta b_{g}$

where $(\cdot {)}^{\wedge }$ and $[\cdot {]}_{\times }$ are equivalent, both representing the isomorphic mapping operator from a 3D vector to a 3 × 3 skew-symmetric matrix. Its significance lies in mapping the uncertainty of attitudes to a Gaussian distribution defined in the three-dimensional vector space, which makes the physical meaning of the covariance matrix P more explicit.

**Table 4:** Computational complexity comparison between the proposed and existing algorithms.

Algorithmic Architecture	Mathematical Mechanism	Computational Complexity of State Estimation	Computational Complexity of Query	Features and Advantages
Standard EKF	First-order linear propagation	$O(m\cdot M^{2}+M^{3})$	N/A	Extremely lightweight, prone to divergence
FGO (Factor Graph)	Sliding window optimization	$O(N_{w}(m\cdot M^{2}+M^{3}))$	$O(N_{p}\log N_{p})$	High accuracy, heavy computational overhead
FAST-LIO2	iESKF + ikd-Tree	$O(K(m\cdot M^{2}+M^{3}))$	$O(\log N_{p})$	Efficiency balance, weak capability in handling skidding
Proposed	Adaptive iESKF + i-Octree	$O(K(m\cdot M^{2}+M^{3}))$	$O(\log N_{p})$	Balances accuracy and robustness

**Table 5:** Honest runtime breakdown of per-component operations (Average per frame).

Algorithmic Module	Average Execution Time (ms)	Percentage of Total Runtime
IMU Pre-integration & Prior State Propagation	0.9	7.0%
Spatial Map Query (i-Octree Search) & Feature Association	4.1	32.0%
Jacobian Construction & Point-wise Mahalanobis Check	2.8	21.9%
Adaptive M-Estimation Weighting (Robust Module)	0.6	4.7%
iESKF Iterative State Update (State-space matrix inversion)	3.2	25.0%
Map Incremental Maintenance (i-Octree Update)	1.2	9.4%
Total Frame Processing Time	12.8	100%

**Table 6:** Quantitative Absolute Trajectory Error (ATE) under structured scenarios.

Algorithm	Translation RMSE (m)	Rotation RMSE (°)
Ours (AR-iESKF)	0.12	0.85
FAST-LIO2	0.15	0.92
FAST-LIVO	0.13	0.88
LVI-SAM	0.11	0.79

**Table 7:** Quantitative comparison of closed-loop translation errors and *Z*-axis drift.

Algorithm	Closed-Loop Translation Error (m)	Maximum *Z*-Axis Drift (m)
Ours (Full AR-iESKF)	1.24	0.32
Naïve Wheel-LVI (Ablation Baseline)	2.84	1.45
FAST-LIO2	2.78	1.87
LVI-SAM (FGO)	11.70	21.45

Note: The values in Table 7 represent the statistical averages of multiple runs, while the indicators highlighted in Figure 3 correspond to a single representative evaluation trial. The Naïve Wheel-LVI baseline incorporates the exact same multi-modal sensors as our proposed AR-iESKF but rigidly fuses them without the adaptive robust mechanisms, highlighting the catastrophic effect of trusting slipping wheels without robust isolation.

**Table 8:** Quantitative performance comparison in challenging industrial scenarios.

Algorithm	End-to-End Drift (m)	Max *Z*-Axis Jitter (m)
Ours (AR-iESKF)	0.35	0.12
FAST-LIO2	0.76	0.26
LVI-SAM (FGO)	1.12	0.41

**Table 9:** Quantitative statistical results of single-frame execution time for each algorithm.

Algorithm	Average Time Consumption (ms)	Median Time Consumption (ms)	99th Percentile Latency (ms)	Real-Time Level
Ours	12.8	12.2	17.9	Near 100 Hz
FAST-LIO2	16.5	15.9	24.6	Meets 50 Hz
FAST-LIVO	23.1	21.8	32.5	Meets 50 Hz
LVI-SAM	41.2	37.5	82.7	Meets 20 Hz

## Data Availability

The original contributions presented in this study are included in the article. Further inquiries can be directed to the corresponding author.
